# Transformation of the endochitinase gene *Chi67*-*1* in *Clonostachys rosea* 67-1 increases its biocontrol activity against *Sclerotinia sclerotiorum*

**DOI:** 10.1186/s13568-016-0313-x

**Published:** 2017-01-03

**Authors:** Zhan-Bin Sun, Man-Hong Sun, Mo Zhou, Shi-Dong Li

**Affiliations:** Institute of Plant Protection, Chinese Academy of Agricultural Sciences, No. 2 West Yuanmingyuan Road, Haidian District, Beijing, 100193 China

**Keywords:** *Clonostachys rosea*, Endochitinase, Mycoparasitism, *Sclerotinia sclerotiorum*, Protoplast transformation, Biocontrol agent

## Abstract

*Clonostachys rosea* is a promising biocontrol fungus active against various plant fungal pathogens. In this study, the endochitinase-encoding gene *Chi67*-*1*, the expression of which is sharply upregulated in *C. rosea* 67-1 when induced by sclerotia, was transformed into the original isolate by protoplast transformation, and transformants were screened against Sclerotinia rot of soybean. The transformation efficiency was approximately 50 transformants per 1 × 10^7^ protoplasts, and 68 stably heritable recombinants were assayed. The parasitic rates of 32.4% of the tested strains increased by more than 50% compared to 43.3% of the wild type strain in 16 h, and the Rc4-4 transformant showed a parasitic rate of 100% in 16 h. The control efficiencies of the selected efficient transformants to soybean Sclerotinia stem rot were evaluated in pots in the greenhouse, and the results revealed that Rc4-4 achieved the highest efficiency of 81.4%, which was 31.7% and 28.7% higher than the control achieved by the wide type and the pesticide carbendazim, respectively. Furthermore, the expression level of *Chi67*-*1* was 107-fold higher in Rc4-4 than in the wild type, and accordingly, the chitinase activity of the recombinant increased by 140%. The results lay a foundation for the development of efficient genetically engineered strains of *C. rosea*.

## Introduction

*Clonostachys rosea* (syn. *Gliocladium roseum*) is a widely distributed mycoparasite of many kinds of plant pathogenic fungi and has shown great potential in controlling plant diseases and promoting crop growth (Cota et al. 2008; Keyser et al. 2016; Morandi et al. 2003; Schöneberg et al. 2015). However, the true potential of this organism is yet to be fully realized, although Prestop, a commercial biocontrol agent (BCA) of *C. rosea* f*. catenulate* (syn. *Gliocladium catenulatum*), is currently mass produced and applied to vegetables, herbs and ornamentals in greenhouses and fields (Chatterton and Punja 2009; Gwynn 2014; Punja and Yip 2003; Rahman and Punja 2007). Thus far, problems such as the unstable efficiency of fungal biopesticides in field applications have limited their development, and the pathogenicity of most wild type isolates needs to be improved to achieve a higher control efficiency.

Several mechanisms are involved in the biocontrol process of *C. rosea*, including mycoparasitism, antagonism, competition for nutrients and space, induction of plant resistance (Lahoz et al. 2004; Papavizas 1985; Rodríguez et al. 2011), and the secretion of a series of cell wall-degrading enzymes (Carsolio et al. 1999; Elad and Kapat 1999; Giczey et al. 2001). Of these, extracellular lytic enzymes, especially chitinase, are considered essential for activity against plant pathogens.

Since the 1990s, chitinolytic enzymes from mycoparasites, especially *Trichoderma* spp., have attracted considerable attention due to their antifungal activity, and a series of chitinase-encoding genes that are expressed in media containing chitin or fungal cell wall components have been cloned and functionally analyzed (Carsolio et al. 1994; Lorito et al. 1998; Seidl et al. 2005; Zeilinger et al. 1999). Deletion and interference of the endochitinase-encoding gene affected the biocontrol activity of *Trichoderma* against fungal pathogens (Romao-Dumaresq et al. 2012; Woo et al. 1999). A number of chitinase-encoding genes were also cloned from *C. rosea* in recent years, and their expressions were found to be intensely stimulated by the plant pathogens *Rhizoctonia solani* and *Fusarium culmorum* (Gan et al. 2007; Mamarabadi et al. 2008). Tzelepis et al. (2015) knocked down the endochitinase-encoding gene *chiC2*, and found that deletion of *chiC2* decreased the inhibitory ability of *C. rosea* against *R. solani* and *Botrytis cinerea*.

Fungal chitinase has been successfully applied to suppress various soilborne and foliar diseases and improve plant resistance to fungal pathogens (Carsolio et al. 1994; Lorito et al. 1998). Furthermore, transformation of exogenous or homologous genes from *Trichoderma* also greatly improves the efficiency of biocontrol fungi (Deng et al. 2007; Haran et al. 1993; Limón et al. 1999; Margolles-Clark et al. 1996; Yang et al. 2011). However, few effective *C. rosea* strains have been genetically engineered, and the genetic manipulation of antifungal chitinase genes from mycoparasite *C. rosea* is largely unexplored.

*Clonostachys rosea* strain 67-1 was originally obtained from vegetable soil using a sclerotia baiting method, and this strain exhibits strong biocontrol activity against a range of plant fungal pathogens (Ma et al. 2011; Zhang et al. 2004). Transcriptome sequencing and analysis of 67-1 parasitizing *S. sclerotiorum* sclerotia revealed a remarkable up-regulation of *Chi67*-*1*, which encodes a 37 kDa endochitinase. The presence of this gene suggested that *Chi67*-*1* was correlated with mycoparasitism of *C. rosea* (Sun et al. 2015b). Therefore, in the present study, we investigated the role of this chitinase in mycoparasitism of *C. rosea* against *S. sclerotiorum*, and assessed the efficiency of 67-1 transformants overexpressing *Chi67*-*1* against Sclerotinia rot of soybean, with the intention of improving the biocontrol of *C. rosea*.

## Materials and methods

### Strains and gene

*Clonostachys rosea* 67-1 was originally isolated from a vegetable yard in Ledong farm in Hainan Province, China (Zhang et al. 2004). *S. sclerotiorum* Ss-H was isolated from Sclerotinia stem rot of soybean in Heilongjiang Province. Both isolates were deposited in the Agricultural Culture Collection of China (strain number: ACCC 39160, ACCC 39161).

Endochitinase gene *Chi67*-*1* (GenBank accession number KT985453) was isolated from *C. rosea* 67-1 induced by *S. sclerotiorum* sclerotia. The gene encodes a 37 kDa secreted protein with a chitinase-like domain that belongs to group B of glycoside hydrolase family 18.

### Construction of recombinant plasmid

Plasmid pAN7-1 was used to overexpress the *Chi67*-*1* gene. This plasmid contained a glyceraldehydes-3-phosphate dehydrogenase promoter (*gpdA*), a terminator (*trpC*), a hygromycin B-resistant gene (*hph*), and several restriction enzyme cutting sites. The *gpdA* and *trpC* fragments were amplified using the primers *qdz*F/*qdz*R and *zzz*F/*zzz*R, respectively, with a concentration of 10 μM. The open reading frame (ORF) of *Chi67*-*1* was amplified from the genomic DNA of *C. rosea* 67-1 using the CF and CR primers (Sun et al. 2015c; Table 1).

**Table 1:** Primers used in this study

Primer	Sequence (5′–3′)	Purpose
*Chi*F	GTTGTGGTTTGCCTGGTG	Real-time PCR
*Chi*R	CCGATACTCTGCTGCTCAT	
*hph*F	ATGCCTGAACTCACCGCGACGTCTG	*Hygromycin* amplification
*hph*R	CTATTCCTTTGCCCTCGGACGAGTG	
CF	TTCAGAGTAGGCTTTTGGATTGGT	*Chi67*-*1* amplification
CR	ACCCCATATTTGCTCATAATCACA	
*qdz*F	ACTCGACCTGCAGGCATGCAAGCTTGAATTCCCTTGTATCTCTACACA	*gpdA* amplification
*qdz*R	ACCAATCCAAAAGCCTACTCTGAAGGGAAAAGAAAGAGAAAAGAAAAG	
*zzz*F	TGTGATTATGAGCAAATATGGGGTGGATCCACTTAACGTTACTGAAA	*trpC* amplification
*zzz*R	GTAAACGACGGCCAGTGCCAAGCTTTCGAGTGGAGATGTGGAGTGG	

**Fig. 1:**
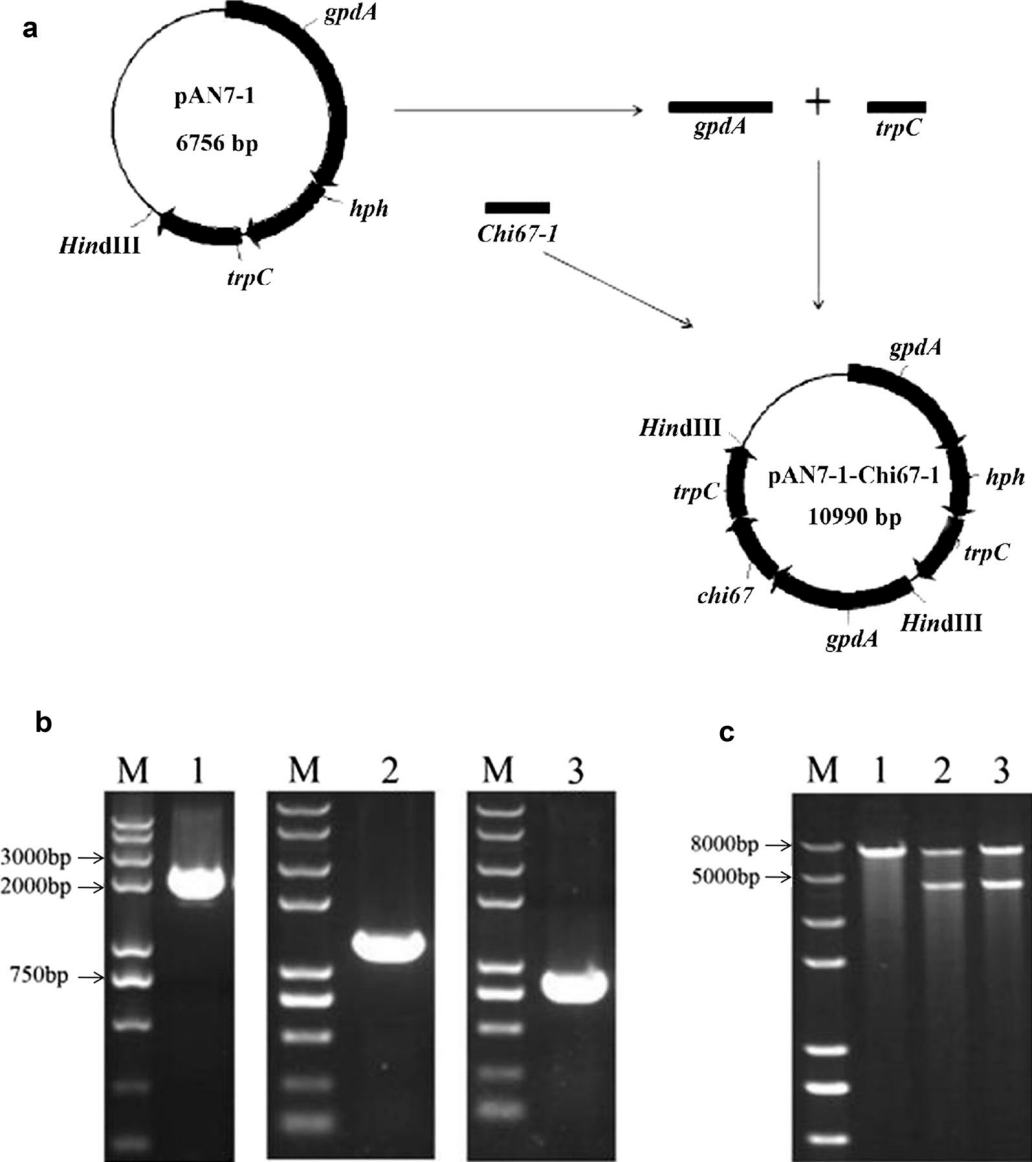
Construction of the transformation vector pAN7-1-Chi67-1. **a** *gpdA* and *trpC* fragments, which were cloned from plasmid pAN7-1, and the *Chi67*-*1* fragment cloned from the *C. rosea* strain 67-1 genome were ligated to the linearized pAN7-1, which was digested with *Hin*dIII to construct the transformation vector pAN7-1-Chi67-1. **b** PCR amplification of inserted fragments. *M* DNA marker; *1 gpdA*; *2 Chi67*-*1*; *3 trpC*. **c** verification of transformation vector. *1* pAN7-1; *2, 3*: constructed vector pAN7-1-Chi67-1

The recombinant plasmid pAN7-1-Chi67-1 was constructed as follows: pAN7-1 was digested with *Hin*dIII, and then ligated together with the fragments *gpdA*, *Chi67*-*1* ORF and *trpC*, successively (Fig. 1a) using a pEASY-Uni Seamless Cloning and Assembly Kit (TransGen Biotech, China). Electrophoresis and sequencing were conducted to verify the recombinant plasmid. Afterwards, the plasmid was transferred into *E. coli* DH5α (Transgen Biotech, Beijing, China) for propagation and extracted for gene transformation.

### Protoplast transformation

*Clonostachys rosea* 67-1 was incubated on potato dextrose agar (PDA) at 26 °C for 10 days. Sterile distilled water (5 mL) was added to the plate and the spores were eluted with a sterile glass spatula and transferred into a 250-mL flask containing PD broth. The fungus was cultured on a fermentation shaker at a speed of 180 rpm at 26 °C. After incubation for 12 h, fresh mycelia were collected using a 125-μm sterile sieve, washed five times with sterile distilled water, and rinsed with 0.7 mol L^−1^ of NaCl to maintain the osmotic pressure. Next, 40 mg mL^−1^ of snail enzyme (XJK Biotech, Beijing, China) was added to hydrolyze the mycelia, and the culture was incubated in a shaker at 28 °C at 100 rpm for 3 h. The released protoplasts were filtered through a sterile 0.22-μm microfiber filter, and the filtrate was centrifuged at 1500×*g* for 10 min. The protoplasts were suspended in STC buffer (200 g of sucrose, 50 mL of 1 mol L^−1^ Tris–HCl pH 8.0, and 5.55 g of CaCl_2_ in 1 L distilled water) and adjusted to a concentration of 10^7^ protoplasts mL^−1^.

A 100 μL suspension of *C. rosea* protoplasts was gently mixed with 20 μg of linearized plasmid pAN7-1-Chi67-1 in a 2 mL Eppendorf tube on ice. After 20 min, 1.25 mL of PTC solution (400 g polyethylene glycol 4000, 10 mL of 1 mol L^−1^ Tris–HCl pH 8.0, and 20 mg of CaCl_2_ in 1 L of distilled water) was added to the transformation system, mixed gently, and incubated at room temperature for 20 min. The mixture was then transferred to TB3 medium (3 g of yeast extract, 3 g of casein acid hydrolysate, and 200 g of sucrose in 1 L of distilled water) and cultivated at 26 °C at a speed of 100 rpm for 16 h. The transformed protoplasts were harvested by centrifuging the suspension at 2500×*g* for 10 min and re-suspending the pellet in 200 μL of STC buffer.

### Stability and PCR validation of the transformants

TB3 medium (15 mL) containing 0.7% low melting-point agarose and 300 μg mL^−1^ of hygromycin B were thoroughly mixed with 200 μL of fungal suspension and poured into a Petri dish. After incubation at 26 °C in the dark for 1 day, 10 mL of fresh hygromycin B-containing medium was poured on the surface of the original one. The colonies that emerged on the upper layer were picked up after 5–7 days of culture and transferred to PDA plates containing 300 μg mL^−1^ of hygromycin B three times in succession. The transformants that were able to grow on resistant plates were considered genetically stable. The colony number of the transformed protoplasts on TB3 plates was counted and the transformation efficiency was calculated.

PCR amplification of *hph* was conducted to verify the transformants. Total RNAs of the isolates were extracted using Trizol reagent (Invitrogen, Carlsbad, USA) according to the manufacturer’s instruction, and cDNAs were synthesized using a cDNA Synthesis Kit (Takara, Dalian, China), from which *hph* fragments were amplified with 10 μM primers *hph*F and *hph*R (Table 1). The following PCR protocol was used: 95 °C for 5 min; 30 cycles of 95 °C for 30 s, 55 °C for 30 s, and 72 °C for 1 min; and an extension step at 72 °C for 10 min.

### Growth and sporulation of transformants

A total of 68 transformants were selected randomly for biological assays. A 0.5 cm diameter agar block of the transformants was placed on the center of a PDA medium-containing 9 cm Petri dish and incubated at 26 °C in the dark. The colony morphology of the mutants was observed after 1 week of culture.

Simultaneously, a fungal block was placed on the surface of a PDA plate covered with a piece of sterile cellophane. After incubation for 1 week, the colony diameter and fresh weight together with the cellophane were measured. The spores were eluted from the fungal mycelia by sterile distilled water and the spores were counted under a microscope (BX41, Olympus, Tokyo, Japan). Three replicates were carried out for each transformant.

### Mycoparasitism against *S. sclerotiorum* sclerotia

Sclerotia of *S. sclerotiorum* were prepared in carrot medium (Zhang et al. 2013), surface sterilized with 1% NaClO for 3 min, and rinsed with sterile distilled water. Excess water was removed using sterile filter paper, and sclerotia were immersed into *C. rosea* suspension containing 1 × 10^7^ spores mL^−1^. After 10 min, sclerotia were retrieved, dried in the shade, and incubated on a piece of wet sterile filter paper in a Petri dish at 26 °C. The number of sclerotia infected by *C. rosea* was counted under a stereo microscope (SMZ-10, Nikon, Tokyo, Japan) at 8, 16, and 20 h, respectively, sclerotia covered with *C. rosea* mycelia were considered parasitized. Parasitic rates of the transformants were calculated, and severity of infection was recorded at 72 h. Sclerotia immersed in sterile distilled water and the spore suspension of the wild type *C. rosea* 67-1 served as controls. A total of 30 sclerotia were tested for each transformant, and three replicates were used.

### Chitinase activity

Highly parasitic transformants were selected for subsequent assays. The 67-1 isolate and its transformants were inoculated into PD broth containing 1% sclerotia powder and cultivated at 28 °C in a rotary shaker at 180 rpm for 4 days. The fermentation liquor was centrifuged at 4 °C for 20 min at 3500×*g*, and crude enzymes were prepared. A standard curve was generated using *N*-acetylglucosamine as a standard, and the chitinase activity of the transformants was determined (Reissig et al. 1995).

### Expression of *Chi67*-*1* in the transformants

The level of transcription of *Chi67*-*1* in the transformants was assayed using an IQ5 Multicolor Real-Time PCR Detection System (Bio-Rad, CA, USA) and SYBR Premix Ex Taq (Takara, Dalian, China), with the elongation factor *EF1* as a reference gene (Sun et al. 2015a). Primers *Chi*F and *Chi*R were designed and synthesized (Table 1), and the specificity was verified by using PCR amplification and sequencing. 10 μM of each primer was used for real-time PCR with the following program: 95 °C for 30 s, followed by 40 cycles of 95 °C for 5 s and 55 °C for 30 s. After PCR, fluorescence values were collected every 0.5 °C from 55 to 95 °C, and the relative expression level of *Chi67*-*1* was calculated using the 2^−ΔΔCt^ method (Livak and Schmittgen 2001). Three replicates were performed for each transformant.

### Control efficacy against soybean Sclerotinia stem rot

Seven transformants with the highest rates of parasitism were selected for evaluation of their ability to control *S. sclerotiorum* on soybean in greenhouse experiments. Soil was collected from an experimental field in the Institute of Plant Protection, Chinese Academy of Agricultural Sciences (CAAS), mixed thoroughly with 20% nursery substrate, and placed in 11 cm plastic pots. A soybean seedling (cultivar *Zigongdongdou*, Institute of Crop Sciences of CAAS, China) was planted in each pot and watered every other day. When six compound leaves were grown, the plants were sprayed with 100 mL of spore suspension of the transformants with a concentration of 5 × 10^6^ spores mL^−1^. After 2 h, leaves were inoculated with an equal volume of *S. sclerotiorum* mycelial suspension. Seedlings treated with the wild type strain followed by the pathogen served as controls, and seedlings treated with the pesticide carbendazim (1000× dilution) were simultaneously assayed. 12 pots were tested for each isolate, and all leaflets on each seedling were determined. Disease severity was recorded using a 9-grade scoring system based on the area of lesions on soybean leaves as follows: 0, no symptoms; 1, <5%; 3, 5–10%; 5, 11–25%; 7, 26–50%; 9, >50%. After 7 days, the disease index was calculated using the formula: disease index = [Σ(number of infected leaves × disease grade)/(total number of leaves × the highest disease grade)] × 100, and the control efficiency of each treatment was evaluated. Three replicates were performed for each sample.

### Statistical analysis

The statistical software SAS 9.1.3 (SAS Institute Inc., Cary, NC, USA) was used for the analysis of variance (ANOVA). Fisher’s LSD test was used to compare the means of fungal growth and sporulation, and Duncan’s multiple range test was used to compare the means of biocontrol activities and gene expression levels. A *P* value <0.05 was considered significant.

## Results

### Construction of pAN7-1-Chi67-1

The target fragments of *gpdA* (2097 bp), *Chi67*-*1* (1307 bp), and *trpC* (770 bp) were successfully detected by electrophoresis (Fig. 1b). Recombinant plasmid pAN7-1-Chi67-1 was digested with *Hin*dIII and verified by electrophoresis, and two bands of the expected size were observed (Fig. 1c). Results of the sequencing indicated successful construction of the recombinant plasmid.

### Screening and biological characterization of transformants

The *Chi67*-*1* gene was transferred into *C. rosea* 67-1 with an efficiency of approximately 50 transformants per 1 × 10^7^ protoplasts. A total of 68 transformants that grew stably on hygromycin-containing plates were verified by PCR amplification of a specific fragment of *hph*, indicating that pAN7-1-Chi67-1 had successfully integrated into the 67-1 strain.

Morphological observation revealed that the colonies of all the transformants and the wild type strain were similar. Of the 68 transformants tested, the largest extension diameter of a colony was 69.5 ± 2.8 mm in 7 days, while the smallest was 61.2 ± 2.2 mm (LSD = 3.4, *P* < 0.05). The colony diameter of 30 transformants was larger than that of the wild strain (63.7 ± 2.7 mm). Sporulation of transformants ranged from (5.3 ± 0.2) × 10^7^ to (57.2 ± 1.6) × 10^7^ spores plate^−1^ (LSD = 2.3 × 10^7^, *P* < 0.05), and more than a third of the mutants exhibited an increased sporulation ability compared with the wild type (17.1 × 10^7^ spores plate^−1^).

### Mycoparasitism of transformants against *S. sclerotiorum* sclerotia

**Fig. 2:**
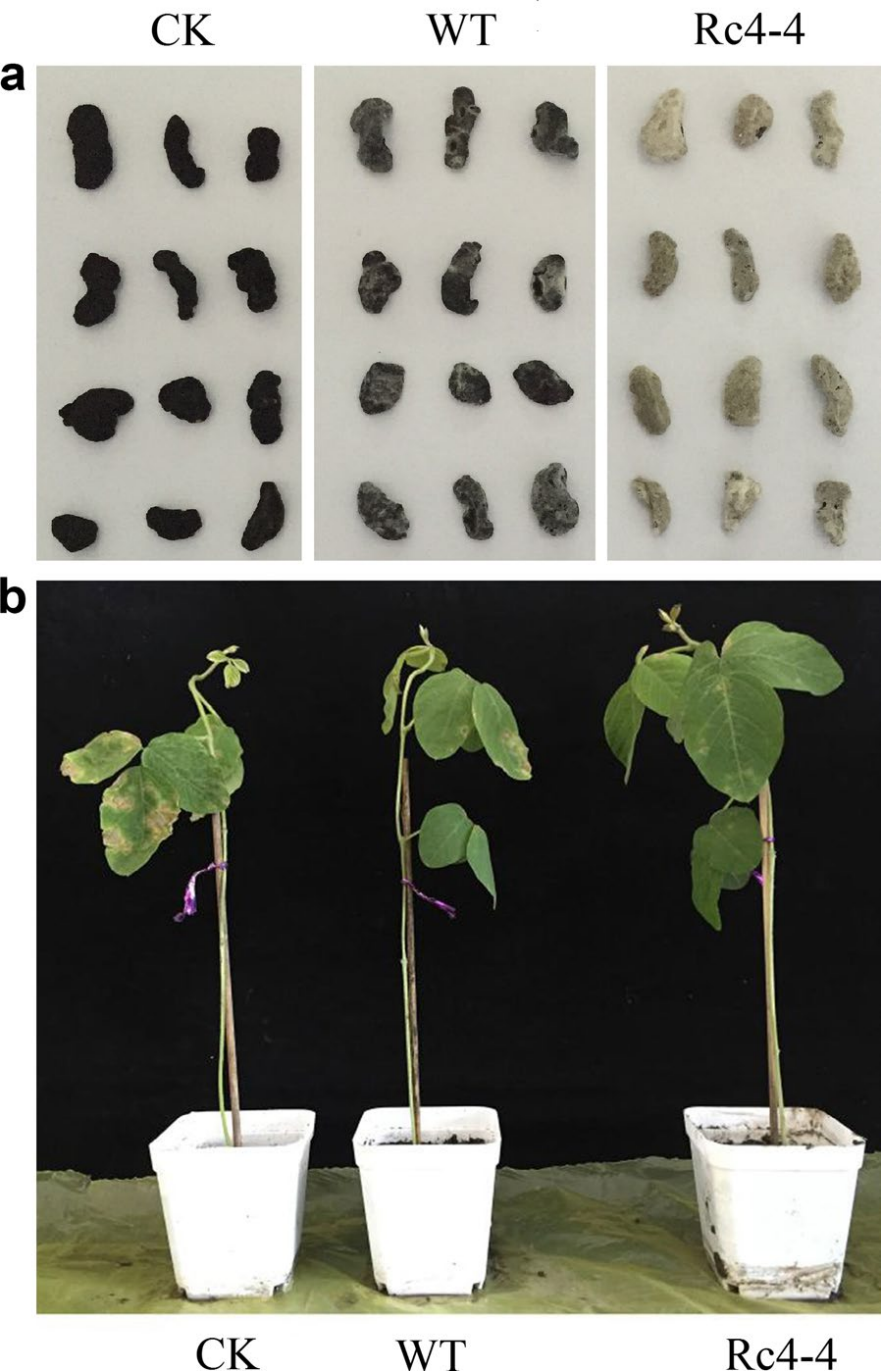
Bioassay of *Chi67*-*1* transformant Rc4-4. **a** mycoparasitism of *C. rosea* 67-1 and Rc4-4 against *S. sclerotiorum* sclerotia at 72 h. **b** control efficiency of *C. rosea* 67-1 and Rc4-4 against soybean Sclerotinia stem rot in pot experiments. *CK* distilled water, *WT* wild type

No hyphae were observed on the surface of the sclerotia at 8 h after inoculation with the wild strain. However, hyphae were visible in some sclerotia inoculated with transformants. For example, Rc5-1 and Rc5-7 treated sclerotia showed mycoparasite hyphae beginning to extend outwards, and by 16 h, mycoparasitism was evident on all sclerotia infected with transformants and the wild type strain, but the parasitic ability of mutants was clearly higher than the wild type (*P* < 0.05). The parasitic rates of nearly a third of the transformants increased by 50%, compared to 43.3% for isolate 67-1 (Table 2). Furthermore, there was a significant increase in mycoparasitism over time, and transformants produced considerably more hyphae that eventually covered the whole sclerotia surface, and infected sclerotia were demonstrably softer and rotten at 72 h (Fig. 2a).

**Table 2:** Parasitic rate of *Chi67*-*1* transformants of *C. rosea* 67-1 against sclerotia of *S. sclerotiorum*

Strain	8 h (%)	16 h (%)	20 h (%)
CK	0.0^b^	0.0^h^	0.0^c^
Rc1-1	0.0^b^	76.7^bcd^	93.3^ab^
Rc2-8	0.0^b^	66.7^cde^	93.3^ab^
Rc2-10	0.0^b^	60.0^def^	90.0^ab^
Rc3-5	0.0^b^	59.3^def^	88.9^ab^
Rc3-7	0.0^b^	53.3^ef^	90.0^ab^
Rc4-4	0.0^b^	100.0^a^	100.0^a^
Rc4-5	0.0^b^	83.3^abc^	100.0^a^
Rc4-7	0.0^b^	86.2^ab^	93.3^ab^
Rc5-7	3.33^a^	50.0^ef^	90.0^ab^
Rc8-5	0.0^b^	30.0^g^	90.0^ab^
WT	0.0^b^	43.3^gf^	83.3^b^

Data are means of three replicates. Values within a column followed by different letters are significantly different at *P* < 0.05 according to Duncan’s multiple range test

*WT* wild type, *CK* distilled water

### Chitinase activity

A standard curve of chitinase activity was generated using the regression equation *y* = 0.0038*x* + 0.0304, with a correlation coefficient (*R* ^2^) of 0.9919. The chitinase activity of transformants was calculated and ranged from 124.1 to 308.8 U mL^−1^. Most of the transformants displayed a higher chitinase activity than the wild strain, and activity was highest for transformant Rc4-4, which was 2.4-fold higher than that of the wild type (Table 3).

**Table 3:** Chitinase activity of *Chi67*-*1* transformants of *C. rosea* 67-1

Strain	Enzyme activities (U mL^−1^)
Rc1-1	124.1^f^
Rc2-8	150.5^de^
Rc2-10	125.1^f^
Rc3-5	257.9^b^
Rc3-7	169.2^cd^
Rc4-4	308.8^a^
Rc4-5	179.5^c^
Rc4-7	181.9^c^
Rc5-7	145.6^ef^
Rc8-5	171.2^cd^
WT	129.3^ef^

Data are means of three replicates. Values within a column followed by different letters are significantly different at *P* < 0.05 according to Duncan’s multiple range test

*WT* wild type

### Transcription level of *Chi67*-*1* in the transformants

**Fig. 3:**
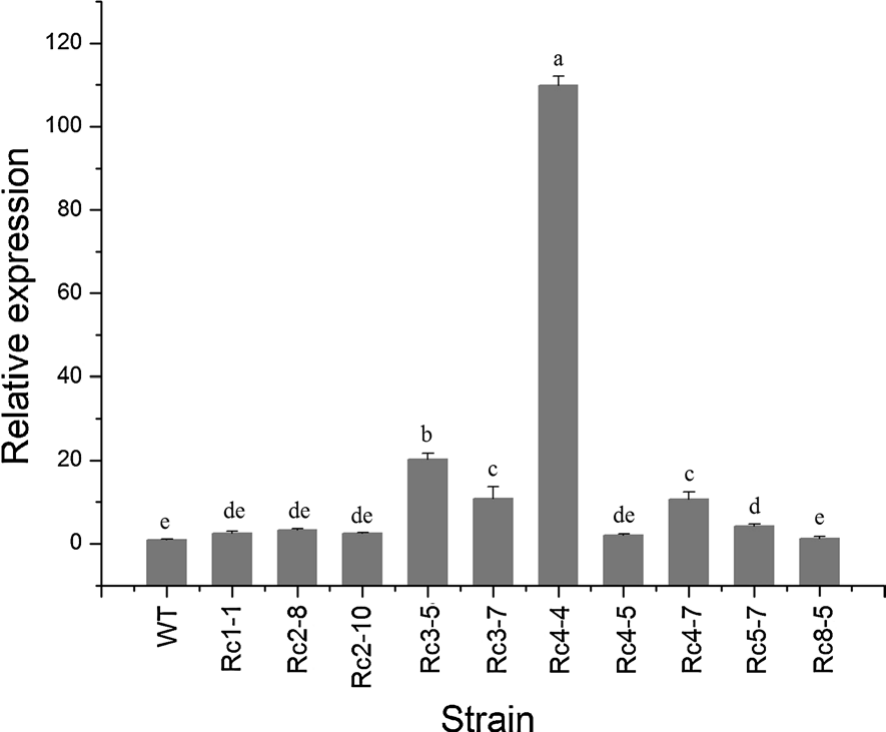
Expression levels of *Chi67*-*1* from *C. rosea* 67-1 and its transformants assessed using quantitative real-time PCR. *WT* wild type. *Error bars* indicate the standard deviations of three replicates. *Different letters* indicate significant differences (*P* < 0.05) according to Duncan’s multiple range test

The expression of *Chi67*-*1* in transformants was increased significantly compared with the wild type strain (*P* < 0.05). Consistent with the result of chitinase activity measurement described above, expression of *Chi67*-*1* was highest in transformant Rc4-4, which was 107-fold higher than the wild type (Fig. 3).

### Control efficacy of *C. rosea* transformants against soybean Sclerotinia stem rot in greenhouse

Soybean leaves exhibited serious infection after incubation with the pathogen for 7 days. However, soybean seedlings treated with both the biocontrol fungi and pesticide were much healthier and exhibited less damage than the control. Consistent with the results of the *Chi67*-*1* expression and chitinase activity measurement described above, the Rc4-4 transformant exhibited the highest control efficiency against soybean Sclerotinia stem rot (81.4%), which was 31.7 and 28.7% higher than seedlings treated with the wild type strain and carbendazim, respectively (Table 4; Fig. 2b). Therefore, transformant Rc4-4 can be considered an efficient agent for controlling Sclerotinia rot of soybean.

**Table 4:** Control efficacy of the *Chi67*-*1* transformants of *C. rosea* 67-1 against soybean Sclerotinia stem rot in pots (7 days)

Strain	Disease index	Control efficacy (%)
Rc1-1	24.4^b^	67.7^b^
Rc2-8	27.4^b^	63.7^b^
Rc2-10	26.7^b^	64.7^b^
Rc3-7	31.1^b^	58.8^b^
Rc4-4	14.1^c^	81.4^a^
Rc4-5	22.2^bc^	70.6^ab^
Rc4-7	25.2^b^	66.7^b^
Carbendazim	27.8^b^	63.2^b^
WT	28.9^b^	61.8^b^
CK	74.8^a^	–

Data are means of three replicates. Values within a column followed by different letters are significantly different at *P* < 0.05 according to Duncan’s multiple range test

*WT* wild type, *CK* distilled water

## Discussion

Biological methods have great potential for controlling plant fungal diseases. In 2015, China proposed a “two-reduction” strategy, i.e. reducing the application of chemical fertilizers and pesticides, which aims to fight against pollution problem in agriculture and improve food safety in China. Biocontrol agents are becoming increasingly important in achieving these goals while simultaneously enhancing crop production. However, some problems such as relatively low efficiency and instability of biopesticides have limited their development so far (Kredics et al. 2003; Slusarski and Pietr 2009). More biocontrol agents with high efficiency will be obtained with genetic improvement. In this study, we overexpressed the endochitinase-encoding gene *Chi67*-*1* in the biocontrol fungus *C. rosea* 67-1 through protoplast transformation, selected several transformed strains with increased *Chi67*-*1* expression, chitinase production and parasitic ability, and evaluated their ability to protect against Sclerotinia rot of soybean in greenhouse experiments. Of the transformants screened in this study, Rc4-4 was found to exhibit a similar colony morphology, growth rate and sporulation ability as the wild strain, but this strain displayed a much higher capacity to control Sclerotinia rot of soybean. To the best of our knowledge, this is the first study to report the construction of a genetically engineered strain of *C. rosea* overexpressing an antifungal chitinase gene in order to enhance the biological control activity.

In a previous study, the endochitinase-encoding gene *Chi67*-*1* was found to be significantly upregulated in *C. rosea* 67-1 parasitizing *S. sclerotiorum*, compared with expression in 67-1 not exposed to *S. sclerotiorum* (Sun et al. 2015b). Therefore, we decided to attempt to genetically engineer a highly efficient biocontrol strain by overexpressing this gene. The strategy and the result were similar to that seen in another study in which overexpression of the chitinase-encoding gene *chit33* from *T. harzianum* CECT 2413 led to an increased inhibition of the growth of *R. solani* (Limón et al. 1999). Importantly, *Chi67*-*1*, which encodes a high-quality cell wall-degrading enzyme, can also be mass produced and utilized in the management of plant fungal diseases.

In vitro assays showed that most transformants exhibited a higher parasitic ability against sclerotia compared to the wild type strain. However, in pot experiments, only one transformant, Rc4-4, exhibited a much higher ability to control soybean Sclerotinia stem rot. This might be due to various antagonistic mechanisms other than a single pattern of mycoparasitism. The management of plant diseases with *C. rosea* involves several mechanisms such as mycoparasitism, antagonist action, induced resistance, and competition, which enhance the performance of the biocontrol fungus (Mouekouba et al. 2014; Zhang et al. 2008). In addition, colonization of the biocontrol isolates on plants during the growing season can also greatly affect the control efficiency (Vallance et al. 2009).

Of the 68 *Chi67*-*1* transformants tested in this study, only a few obvious differences in colony morphology were observed. However, the transformants exhibited a wide variation in sporulation, ranging from 5.3 to 57.2 × 10^7^ spores plate^−1^. This may be due to a random insertion of the recombinant plasmid that may have affected the sporulation pathway of the transformants and modified the expression of sporulation-related genes (Park et al. 2013; Yu et al. 2015). Since a high sporulation ability facilitates fungal mass production and commercialization, this may be a desirable feature in fungal strains to be used as biocontrol agents. Further studies should therefore be carried out to determine the safety of this *C. rosea* transformant and further assess its capacity to control soybean Sclerotinia stem rot.

## Abbreviations

*Hph*: hygromycin B-resistant gene
ORF: open reading frame
PDA: potato dextrose agar
PD: potato dextrose broth
CAAS: Chinese Academy of Agricultural Sciences
CK: control
WT: wild type
